# Factors for inhibition of early discharge from the psychiatric emergency ward for elderly patients

**DOI:** 10.1186/s12199-018-0738-8

**Published:** 2018-10-10

**Authors:** Sho Adachi, Tomoko Komiya, Kenji Nomura, Masayuki Shima

**Affiliations:** 1 Department of Psychiatry, Arimakougen Hospital, Kobe, Japan; 2 Department of Public Health, Hyogo College of Medicine, 1-1 Mukogawa-cho, Nishinomiya, 663-8501 Japan

**Keywords:** Hypoalbuminemia, Body mass index, C-reactive protein, Elderly psychiatric patients, Dementia, Early discharge, Psychiatric emergency ward

## Abstract

**Background:**

As society is aging, the number of elderly patients with psychiatric disorder, such as dementia, is increasing. The hospitalization period of elderly patients in psychiatric wards tends to be prolonged. In this study, we have determined the factors that inhibit early discharge from the psychiatric emergency ward for elderly patients in Japan.

**Methods:**

The information was collected from patients admitted to our hospital’s emergency ward for elderly patients with psychiatric disorders between May 2015 and April 2016. We compared various factors between the early discharge group and the non-early discharge group. In addition, we used a multiple logistic regression model to clarify the risk factors for non-early discharge.

**Results:**

Of the 208 elderly patients, body mass index (BMI) and serum albumin level were significantly lower in the non-early discharge group. In addition, we also showed that higher serum C-reactive protein (CRP) (> 0.5 mg/dL) and use of seclusion or physical restraint significantly inhibited the early discharge of patients. The results of multiple logistic analysis showed that the BMI ≤ 17.5 kg/m^2^ (OR, 2.41 [95% confidence interval (CI) 1.06–5.46]), serum albumin level ≤ 30 g/L (OR, 3.78 [95% CI 1.28–11.16]), and use of seclusion or physical restraint (OR 3.78 [95% CI 1.53–9.37]) are particularly important explanatory factors.

**Conclusions:**

Hypoalbuminemia, low BMI, and the use of seclusion or physical restraint were identified as the factors that inhibit early discharge from the psychiatric emergency ward for elderly patients. These factors reflect malnutrition and extremely serious psychiatric symptoms.

## Background

The ration of elderly people in Japan was 26.7% in 2015, which is extremely high compared with the global elderly ration of 8.3%, and Japan is the world’s most rapidly aging country [1]. As the elderly population is growing, the number of elderly patients with psychiatric disorders, including dementia, is also increasing [2]. In 2012, there were 4.62 million dementia patients, meaning that 1 in every 7 elderly people over 65 years old had dementia. In 2025, it is expected that the number of dementia patients will increase to about 7 million, meaning that 1 in every 5 elderly people will have dementia [1]. Currently, there are 47 million dementia patients worldwide, and the number of patients is expected to increase to 131 million by 2050 [3].

In addition to the core symptoms such as impaired memory and disorientation, the behavioral and psychological symptoms of dementia (BPSD) including psychomotor excitation, delusions, and increased irritability become active in patients with dementia [4]. There are numbers of reports on the prevalence rate of BPSD in various countries. Ikeda et al. reported that the prevalence rate of BPSD was 85% in Japan, which was very high [5]. Because patients with BPSD require psychiatric therapies, many patients have to be hospitalized [6, 7]. In addition, there are various psychiatric disorders in old age including mood disorders, alcohol-related disorders, and schizophrenia. Approximately 15% of adults aged 60 and over suffer from a mental disorder [2].

In Japan, about 70% of patients are discharged within 3 months from general psychiatric wards. In dementia wards, however, only 40% or less of patients can be discharged within 3 months [8]. Yoshie et al. reported that the average length of hospitalization for the treatment of BPSD in 27 dementia wards was 355.2 ± 216.6 days [9]. Shinjo et al. reported that older age is one of the significant factors associated with prolonged length of stay in psychiatric patients in Japan [10]. There were also reports that elderly patients with psychiatric disorders and dementia are hospitalized for a long period in other countries [11, 12]. Previous studies reported that incompetence, positive symptoms, and low pensions inhibit early discharge of patients from hospitals in the elderly psychiatry field [13, 14]. However, there have been few studies conducted in this field, and the factors on which we can easily intervene have not been fully identified. Therefore, we hypothesized that there were factors inhibiting early discharge of elderly patients with psychiatric disorders from emergency ward and conducted the present study to investigate these factors.

## Methods

### Patients

In Japan, there are few psychiatric emergency wards for elderly people. This study was conducted at one of the wards. This ward generally accepts emergency patients with psychiatric disorders aged 65 or over 24 h a day, 365 days a year. All patients admitted to this emergency ward between May 2015 and April 2016 were sequentially registered to this study, and their medical records were collected. At the time of admission, chest X-ray, head CT, biochemical examination, urinalysis, and infectious disease tests were routinely performed for these patients and ascertained whether patients had medical diseases. We did not hospitalize patients with symptoms of infection such as fever and had them visit an internal medicine department. The hospitalized patients received medication, psychotherapy, and occupational therapy according to the judgment of the primary doctor for each patient. Patients with severe malnutrition received intervention from nutrition support team composed of medical doctor, nurse, and dietician. The group of patients who returned home or returned to the facility after discharge within 3 months was defined as the “early discharge group.” By contrast, we defined the group of patients who were hospitalized for more than 3 months, were transferred to another hospital, or died within 3 months as the “non-early discharge group.”

### Clinical assessment

The following information was collected from the patients at the time of admission. Age, gender, diagnosis, height, weight, body mass index (BMI), serum albumin (ALB) level, C-reactive protein (CRP) level, and hemoglobin (Hb) concentration. We also examined whether or not the patients were involuntarily hospitalized, had a treatment history for the disease, had a history of psychiatric hospitalization, had diabetes or hypertension, had mastication or swallowing dysfunction, and could walk independently. In addition, we reviewed the results of psychological examinations (Neuropsychiatric Inventory (NPI) [15], Revised Hasegawa Dementia Scale (HDS-R) [16], and Mini-mental State Examination (MMSE) [17]). HDS-R and MMSE share several question items and there is a high correlation between them. These examinations were performed for the same patient group in a previous study. The results showed that the areas under the curve (AUC) of HDS-R and MMSE were 0.952 and 0.902, respectively. The value from HDS-R was higher than that from MMSE, indicating that the HDS-R is more accurate than the MMSE in diagnosing Alzheimer’s disease [16].

Furthermore, the following information was collected from the patients during hospitalization. The number of times of daily oral administration, the use of drugs (antipsychotic drugs, anti-dementia drugs, and Yokukansan [18]), and use of seclusion or physical restraint. Yokukansan (Yi-Gan San): The results of the randomized controlled trial showed that Yokukansan significantly improved BPSD [18]. The diagnosis was made according to ICD-10 (International Classification of Diseases, Tenth Revision).

### Statistical analysis

We compared each factor using a *t* test or chi-square test between the early discharge group and non-early discharge group, determining which risk factors inhibit early discharge based on the results of the comparison. Besides, we set the cutoff values to BMI ≤ 17.5 kg/m^2^ and serum ALB ≤ 30 g/L and compared the percentages between the groups using a chi-square test. It has been shown that a high morbidity was associated with a very low BMI, especially a BMI < 17.5 kg/m^2^ [19]. According to the CONUT (a tool for controlling nutritional status), ALB ≤ 30 g/L is one of the indicators of moderate malnutrition [20].

In addition, a multiple logistic regression analysis was used to clarify the risk factors for non-early discharge, after adjusting age, BMI, serum ALB, serum CRP, and use of seclusion or physical restraint. In accordance with the standard method for multiple logistic analyses, the required number of outcomes was set to 10 or more for each independent variable.

Next, the patients were divided into four groups by the number of risk factors for each patient, and then the trend of the percentages of early discharge among the groups was estimated using the Cochran–Armitage trend test. To investigate the association between the number of risk factors and non-early discharge, a multiple logistic regression analysis was performed, after adjusting age, the number of risk factors, and use of seclusion or restraint.

The significance level of all statistical analyses was *p* < 0.05. All statistical analyses were conducted using R software (ver. 3.3.3).

This protocol received approval from the Ethics Committee of Arimakougen Hospital (Approval number: 2016-07), and it conformed to the provisions of the Declaration of Helsinki. The requirement for written informed consent was waived by the Ethics Committee, since the study involved record review only. The information about this study was posted in the hospital, and patients were given opportunity to opt-out of participation.

## Results

Between May 2015 and April 2016, 213 patients were hospitalized in the psychiatric emergency ward. Five exceptionally hospitalized patients were excluded from the study because they were not elderly and just temporarily hospitalized as an emergency response. Of the 208 elderly patients, 153 (73.6%) patients discharged within 3 months.

Among the 208 patients, there were 156 dementia patients including alcoholic dementia patients (ICD-10 code: F 107). There were 86 patients with Alzheimer type dementia (55.1%), 16 with vascular dementia (10.3%), 16 with Lewy body dementia (10.3%), and 11 with mild cognitive impairment (7.1%). Diagnosis and number of patients are summarized in Table 1.

**Table 1:** Diagnosis and number of patients

ICD-10	*N* (%)
Total	208 (100.0%)
F0	154 (74.0%)
Dementia in Alzheimer disease	86 (41.3%)
Vascular dementia	16 (7.7%)
Dementia with Lewy bodies	16 (7.7%)
Mild cognitive impairment(MCI)	11 (5.3%)
F1	12 (5.8%)
Alcohol-related dementia	6 (2.9%)
F2	13 (6.3%)
F3	20 (9.6%)
Major depressive disorder	16 (7.7%)
Bipolar disorder	4 (1.9%)
F4	5 (2.4%)
F7	1 (0.5%)
F8	1 (0.5%)
G20	2 (1.0%)

*F0* Organic, including symptomatic, mental disorders, *F1* Mental and behavioral disorders due to psychoactive substance use, *F2* Schizophrenia, schizotypal and delusional disorders, *F3* Mood [affective] disorders, *F4* Neurotic, stress-related and somatoform disorders, *F7* Mental retardation, *F8* Disorders of psychological development, *F9* Behavioral and emotional disorders with onset usually occurring in childhood and adolescence, *G20* Parkinson’s disease

The BMIs of the patients at the time of admission were 21.0 ± 3.5 (kg/m^2^) and 19.7 ± 3.6 (kg/m^2^) in the early discharge group and non-early discharge group, respectively. The BMI was significantly lower in the non-early discharge group than that in the early discharge group (*p* = 0.029). The percentage of patients with BMI ≤ 17.5 kg/m^2^ was also lower in the early discharge group than that in the non-early discharge group (*p* = 0.011). The serum ALB level at the time of admission was significantly lower in the non-early discharge group than that in the early discharge group (*p* = 0.009). The percentage of patients with ALB ≤ 30 g/L was also significantly lower in the early discharge group than that of patients in the non-early discharge group (*p* = 0.004). At the time of admission, the percentage of patients with serum CRP > 0.5 mg/dL in the early discharge group was significantly lower than that of patients in the non-early discharge group (*p* = 0.049). The percentage of patients for whom seclusion or physical restraint was used during hospitalization in the early discharge group was significantly lower than that of patients in the non-early discharge group (*p* < 0.001). There were no significant differences in other examined factors (Table 2).

**Table 2:** Comparison of each factor between the early discharge group and the non-early discharge group

	Early discharge (*n* = 153)	Non-early discharge (*n* = 55)	*p* value
Characteristics at admission			
Age ≤ 75 years old	41 (26.8%)	20 (36.4%)	0.245
Gender: male	56 (36.6%)	24 (43.6%)	0.448
Height (cm) (N = 201)	153.5 ± 10.2	155.3 ± 9.3	0.272
Weight (kg) (*N* = 203)	49.6 ± 10.3	47.8 ± 10.1	0.276
BMI (kg/m^2^) (*N* = 201)	21.0 ± 3.5	19.7 ± 3.6	0.029
BMI ≤ 17.5 kg/m^2^ (*N* = 201)	21 (14.0%)	16 (31.4%)	0.011
Biochemical examination			
Serum albumin (g/L)	37.3 ± 4.2	35.4 ± 5.6	0.009
Serum albumin ≤ 30 g/L	10 (6.5%)	12 (21.8%)	0.004
Serum C reactive protein, > 0.5 mg/dL	69 (45.1%)	34 (61.8%)	0.049
Hemoglobin (g/L)	122.4 ± 15.4	123.0 ± 19.4	0.820
Involuntary admission	121 (79.1%)	45 (81.8%)	0.813
History of treatment	133 (86.9%)	46 (83.6%)	0.706
History of hospitalization	37 (24.2%)	11 (20.0%)	0.656
Dementia	112 (73.2%)	44 (80.0%)	0.414
Complications			
Diabetes	27 (17.6%)	9 (16.4%)	0.994
Hypertension	69 (45.1%)	20 (36.4%)	0.704
Mastication or swallowing dysfunction	113 (73.9%)	47 (85.5%)	0.118
Unable to walk independently	50 (32.7%)	24 (43.6%)	0.205
HDS-R (*N* = 164), ≤ 15	74 (61.2%)	28 (65.1%)	0.782
MMSE (*N* = 85), ≤ 12	30 (48.4%)	8 (42.1%)	0.828
NPI (*N* = 124), > 5	66 (71.7%)	19 (59.4%)	0.282
Characteristics during hospitalization			
Use of antipsychotic drugs	96 (62.7%)	30 (54.5%)	0.365
Use of anti-dementia drug	38 (24.8%)	10 (18.2%)	0.413
Use of Yokukansan	50 (32.7%)	17 (30.9%)	0.942
Times of medication per day, ≥ 4	95 (62.1%)	31 (56.4%)	0.559
Use of seclusion or physical restraint	12 (7.8%)	15 (27.3%)	< 0.001

To clarify the risk factors for the non-early discharge group after adjusting the related factors, a multiple logistic regression analysis was performed. Based on the above results, serum ALB ≤ 30 g/L, serum CRP > 0.5 mg/dL, BMI ≤ 17.5 kg/m^2^, and use of seclusion or physical restraint were chosen as explanatory variables. We used five factors including the above four factors and age ≤ 75 years old for analysis by the forced entry method. The odds ratios (ORs) of “non-early discharge” were significant for BMI ≤ 17.5 kg/m^2^ (OR, 2.41 [95% confidence interval (CI) 1.06–5.46]), serum ALB ≤ 30 g/L (OR, 3.78 [95% CI 1.28–11.16]), and use of seclusion or physical restraint (OR, 3.78 [95% CI 1.53–9.37]) (Table 3).

**Table 3:** Odds ratios (OR) and 95% confidence intervals (95% CI) for various factors associated with non-early discharge

	Non-early discharge		
	OR	(95% CI)	*p* value
Factors			
Age ≤ 75 years old	1.89	(0.91–3.94)	0.087
BMI ≤ 17.5 kg/m^2^	2.41	(1.06–5.46)	0.036
Serum albumin ≤ 30 g/L	3.78	(1.28–11.16)	0.016
Serum CRP > 0.5 mg/dL	1.43	(0.70–2.92)	0.322
Use of seclusion or physical restraint	3.78	(1.53–9.37)	0.004

Odds ratios were adjusted for the above five factors

**Fig. 1:**
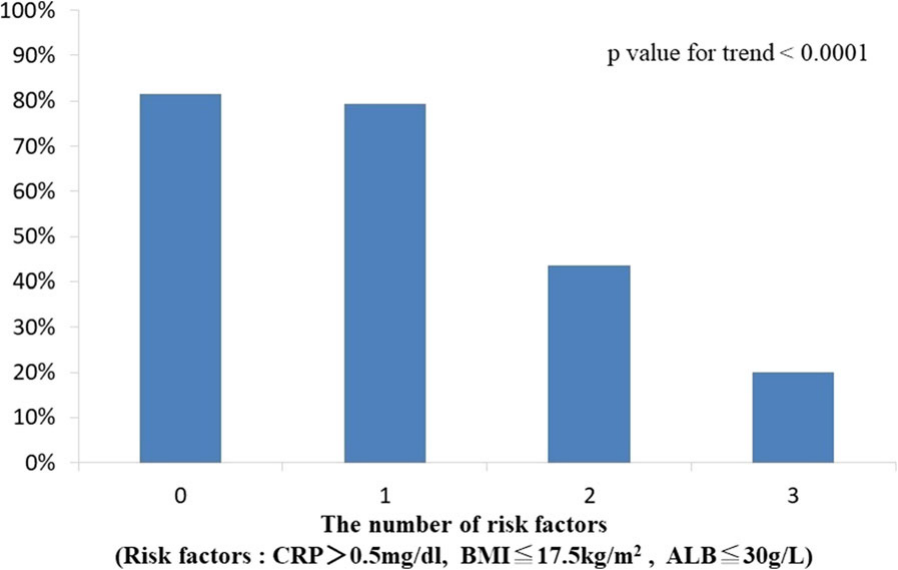
The percentages of early discharge in relation to the number of risk factors

In univariate analysis, BMI ≤ 17.5 kg/m^2^, serum ALB ≤ 30 g/L, and serum CRP > 0.5 mg/dL at the time of admission were significantly associated with inhibition of early discharge. According to the number of these risk factors for each patient, we divided patients into four groups as follows. Valid measurements were obtained from 201 hospitalized patients. Among them, 81 patients had no risk factor, 92 had one risk factor, 23 had two risk factors, and 5 had three risk factors. The percentages of early discharges of these groups were 81.5%, 79.3%, 43.5%, and 20.0%, respectively (*P* value for trend < 0.001) (Fig. 1).

A multiple logistic regression analysis was performed using factors including age ≤ 75 years old, the number of risk factors, and use of seclusion or physical restraint for analysis by the forced entry method. The odds ratios (ORs) of “non-early discharge” were significant for two risk factors (OR, 5.11 [95% CI 1.81–14.40]), three risk factors (OR, 11.63 [95% CI 1.06–127.55]), and use of seclusion or physical restraint (OR, 4.11 [95% CI 1.63–10.36]) (Table 4). The more the number of risk factors, the more the odds ratio was rising.

**Table 4:** Odds ratios (OR) and 95% confidence intervals (95% CI) for various factors associated with non-early discharge

	Non-early discharge		
	OR	(95% CI)	*p* value
Factors			
Age ≦ 75 years old	1.82	(0.87–3.83)	0.213
The number of risk factors			
0	1.00	–	–
1	1.05	(0.48–2.29)	0.863
2	5.11	(1.81–14.40)	0.002
3	11.63	(1.06–127.55)	0.041
Use of seclusion or restraint	4.11	(1.63–10.36)	0.004

Odds ratios were adjusted for the above three factors

## Discussion

In this study, we determined the factors that inhibit early discharge of elderly patients with psychiatric disorders from a psychiatric emergency ward in Japan. During the study period, 73.6% of the elderly patients were discharged within 3 months. The hospitalized patients were mainly dementia patients with behavioral psychological symptoms, and they were discharged earlier than patients in other dementia wards in Japan [8]. However, there were a group of patients who were not discharged within 3 months. We examined this patient group in detail. Ismail et al. previously reported that incompetence and positive symptoms inhibit early discharge of psychiatric elderly patients [13]. Sugiyama et al. reported that a low pension is a factor that inhibits early discharge of some dementia patients [14]. Our study showed that dementia, severity of dementia (determined from the results of HDS-R or MMSE), and intensity of behavioral psychological symptoms (determined by NPI) were not factors that significantly inhibited early discharge, which is similar to the previous studies [13, 14].

The present study showed that several physical factors significantly prolonged the hospitalization period, although the main purpose of hospitalization for the patients was the treatment of psychiatric disorders. The percentages of early discharge were lower in patients with low BMI and serum ALB level. Low BMI is an indicator of malnutrition [21]. BMI has been identified as a predictor for activities of daily life (ADL) [22, 23], and it has been also known to be associated with elderly people’s mortality rate a year later [24, 25]. Inamura et al. reported that numbers of patients with schizophrenia who are hospitalized for 3 months or more have low BMI [26]. Serum ALB level is known to decline as age rises [27–30]. A decline in serum ALB level results in a decrease in muscle mass [29], which is associated with lower ADL [31]. Low serum ALB level has been identified as a factor that inhibits early discharge of patients hospitalized for rehabilitation purposes [31] and for percutaneous endoscopic gastrostomy (PEG) [32]. The low BMI and hypoalbuminemia are thought to result in fraility and sarcopenia and decrease the ADL of patients, which may inhibit early discharge. Haga et al. reported that low BMI and hypoalbuminemia are risk indicators for mortality among psychiatric patients with medical comorbidities [33]. The result was comparable to ours, but we did not investigate the relationship with the mortality rate in this study.

On the other hand, independent walking disability and mastication or swallowing dysfunction did not significantly inhibit early discharge in this study. We showed that higher serum CRP (> 0.5 mg/dL) significantly inhibits early discharge of patients. As mentioned above, since patients who had signs of infection such as fever at the time of admission visited the internal medicine department, these patients were excluded from this study. The causes of the latent inflammatory responses in the patients with serum CRP > 0.5 mg/dL were thought to be chronic infections [34], malignant tumors [35], latent aspiration, or autoimmune responses [34]. Further investigation on this is required.

The patients who required seclusion or physical restraint tended to stay in the hospital for a longer period, although the seclusion or physical restraint was performed after admission. Generally, patients with active problem behavior such as excitement, insult, and violence require seclusion or physical restraint. Therefore, our results suggest that patients with serious symptoms such as excitement, insult, and violence tend to stay in hospital for a longer period.

The results of the multiple logistic analysis showed that the use of seclusion or physical restraint and the low serum ALB level are particularly important explanatory factors. Since seclusion or physical restraint is used for patients due to psychiatric symptoms and problem behavior, it is difficult to reduce the frequency of use. Although patients with severe malnutrition received intervention from the nutrition support team, low serum ALB level at the time of admission was the important factor for inhibition of early discharge. One reason may be that it takes time to improve malnutrition. It is necessary to investigate if the percentage of early discharge rises by more actively intervening to improve malnutrition. In addition, to prevent prolonged stay in hospital, the preventive treatment for malnutrition before admission is considered important. Further studies are needed in order to confirm this hypothesis. Although serum CRP level significantly inhibited early discharge of patients in a univariate analysis, the multiple logistic analyses revealed that the relationship between serum CRP level and early discharge was not significant. This suggests that nutritional status may be associated with the prolongation of the hospitalization period rather than inflammatory responses.

In addition, we focused on the number of risk factors at the time of admission. We regarded ALB ≤ 30 g/L, serum CRP > 0.5 mg/dL, and BMI ≤ 17.5 kg/m^2^ at the time of admission as the risk factors that inhibit early discharge of elderly patients. The percentages of early discharge were significantly lowered with the number of the factors that patients had. Therefore, the possibility of early discharge may be predicted at the time of admission to some extent. In multiple logistic regression model, the more the number of risk factors, the more the odds ratio was rising. This result showed that serum CRP level had no small effect on “early discharge,” although CRP itself was not significant in previous multiple logistic regression model.

In this study, we were able to identify several new physical factors that inhibit early discharge of patients in the elderly psychiatry field. Since these factors can be conveniently measured at the time of admission, the risk for non-early discharge can be estimated at the time of admission to some extent. Furthermore, since intervention is possible for the low serum ALB level and low BMI, it will be necessary to examine whether the intervention promotes early discharge of patients in the future.

There are several limitations in this study. Firstly, there was a problem with the sample size. If more patients had been included in the study, we may have been able to identify more factors that inhibit early discharge. Secondly, the study subjects in this study were selected from patients in only one hospital in a single country. It is unclear whether similar results can be obtained from patients in other hospitals or other countries. The reason is that the medical insurance system, medical facilities, and treatment policy vary depending on the country and hospital. To verify this, further investigation will be required. This study was conducted at one of the few psychiatric emergency wards for elderly patients. The ward was not targeted to patients in a specific and narrow area, so we were able to examine patients from various regions.

## Conclusion

We identified hypoalbuminemia, low BMI, and use of seclusion or physical restraint as factors that inhibited early discharge of patients from a psychiatric emergency ward for elderly patients in Japan. These factors reflect malnutrition and extremely serious psychiatric symptoms. It will be necessary to further investigate whether an intervention, especially for malnutrition, can improve the rate of early discharge.

## Abbreviations

ADL: Activities of daily life
ALB: Albumin
BMI: Body mass index
BPSD: Behavioral and psychological symptoms of dementia
CRP: C-reactive protein
Hb: Hemoglobin
HDS-R: Revised Hasegawa Dementia Scale
MMSE: Mini-mental State Examination
NPI: Neuropsychiatric Inventory
